# Processed Food Consumption and Sleep Quality in School-Aged Children: Insights from a Cross-Sectional Study

**DOI:** 10.3390/nu17020233

**Published:** 2025-01-10

**Authors:** Matilde Sousa Gomes, Juliana Martins, Ana Duarte, Cláudia Augusto, Maria José Silva, Patrícia Padrão, Pedro Moreira, Rafaela Rosário

**Affiliations:** 1 Faculty of Nutrition and Food Sciences, University of Porto, 4150-180 Porto, Portugal; matildesougomes@gmail.com (M.S.G.); patriciapadrao@fcna.up.pt (P.P.); pedromoreira@fcna.up.pt (P.M.); 2 Health Sciences Research Unit: Nursing (UICISA: E), Nursing School of Coimbra, Avenida Bissaya Barreto, Polo C, 3046-851 Coimbra, Portugal; anacspduarte@gmail.com (A.D.); coliveira@ese.uminho.pt (C.A.); mjsilva@ese.uminho.pt (M.J.S.); rrosario@ese.uminho.pt (R.R.); 3 School of Nursing, University of Minho, Edifício 4, Campus de Gualtar, 4710-057 Braga, Portugal; 4 Research Centre on Nursing (CiEnf), School of Nursing, University of Minho, Edifício 4, Campus de Gualtar, 4710-057 Braga, Portugal; 5 Epidemiology Research Unit and Laboratory for Integrative and Translational Research in Population Health, Institute of Public Health, University of Porto, 4050-600 Porto, Portugal

**Keywords:** processed food consumption, NOVA classification, sleep quality, dietary patterns, children’s health

## Abstract

Objective: This cross-sectional study aimed to investigate the association between processed food consumption and sleep quality among school-aged children. Methods: Our sample consisted of 137 children, with 52.6% being girls with a mean age of 7.9 ± 1.2 years. Moreover, 40.2% of children had overweight and 35.9% had experienced sleep problems. Dietary intake was evaluated using two non-consecutive 24 h recalls, and foods were categorized according to the NOVA classification system. The amount of sweet snacks consumed, in grams, was recorded for each meal and throughout the day. Sleep quality was assessed using the Portuguese Children’s Sleep Habits Questionnaire (CSHQ-PT). Results: The study found a significant and positive association between the sleep habits score and daily sweet snack consumption (B = 0.035; 95% CI: 0.011, 0.059). Additionally, there were higher odds of experiencing sleep problems in those children who consumed a higher percentage of energy from ultra-processed foods (OR: 1.043; 95% CI: 1.004, 1.084), higher total daily consumption of sweet snacks (OR: 1.016; 95% CI: 1.006, 1.026), particularly during morning and afternoon snack times (OR: 1.018; 95% CI: 1.002, 1.033). Conclusions: These findings underscore the importance of addressing dietary patterns on children’s sleep quality. Future interventions should account for reducing NOVA 4 and sweet snack consumption in order to improve sleep quality.

## 1. Introduction

Changes in lifestyles in the past few decades have altered dietary patterns, resulting in increased consumption of highly processed foods and a decrease in the intake of fresh and minimally processed foods [1]. In this context, epidemiologists developed the NOVA classification after observing that the rise in cardiovascular diseases, type II diabetes, and anxiety was linked to higher consumption of ultra-processed foods (UPFs) [2]. This classification categorizes foods based on their level of processing into four groups: fresh and minimally processed, processed culinary ingredients, processed foods, and UPFs [3].

Studies from Generation XXI, a Portuguese birth cohort studying early-life health, development, and environmental influences through regular follow-ups, have revealed that UPFs account for 30% of total energy in school-age children [4].

Research shows that Portuguese families with lower incomes face challenges in maintaining healthy eating habits due to financial limitations and limited access to nutritious foods. These poor dietary habits appear to be directly linked to the development of obesity and other non-communicable diseases, and there is increasing concern in Portugal about children’s inadequate diets [4,5].

On the other hand, recent research has started to reveal their potential impact on another crucial aspect of health: sleep, especially in children [6,7].

Sleep plays a crucial role in growth, cognitive development, and overall well-being [8]. Inadequate or poor-quality sleep can result in various adverse outcomes, including impaired learning and memory, heightened risk of obesity, and emotional issues. Parents report that 10–75% of children globally experience sleep difficulties, which can range from temporary benign behavioral problems to more chronic and severe conditions like sleep apnea syndromes [9,10,11]. Given the paramount significance of sleep for children, comprehending the potential disruptors is important [12].

The link between dietary patterns and sleep has been explored, and evidence has revealed that the consumption of ultra-processed foods appears to be associated with adverse sleep-related outcomes [1,13]. This relationship can be partially explained by the characteristics of ultra-processed foods, including their high inflammatory potential, low vitamin content, and elevated glycemic load, which may contribute to increased wakefulness and decreased sleep efficiency in children [14,15]. Furthermore, poor sleep quality has been linked to higher sugar intake, the consumption of energy-dense foods, and an overall lower dietary quality [1].

Recognizing the importance of processed food consumption and sleep in children’s health, it is essential to evaluate the relationship between these two parameters. However, the ultra-processed food category is large, and some foods may be more harmful than others.

Until now, there is scant evidence about the associations between UPFs and sleep outcomes in school-aged children, especially those from vulnerable conditions. Therefore, this study aimed to investigate the association between processed food consumption and sleep quality among school-aged children, looking also at the contribution of sweet snacks.

## 2. Materials and Methods

### 2.1. Study Design

This is a cross-sectional study that used a sub-sample of the BeE-school project, which involved primary schools from regions facing economic and social challenges, including poverty and social exclusion, making them particularly vulnerable.

### 2.2. Dietary Assessment

The dietary intake information was collected by trained researchers through two non-consecutive 24 h recalls and did not include non-typical days, such as weekends. Children provided detailed descriptions of their eating occasions, including the time and place of consumption, a detailed description of the food or beverages, the quantity consumed, and the brand of manufactured foods.

A picture booklet was used to identify portion sizes and the Weights and Portions Manual [16] was used to estimate them. Energy and nutrient intake were estimated using the Food Processor Software (Version 11.14). If nutritional information was not available, it was obtained from food labels on supermarket sites.

To address misreporters, we exclude those with daily energy intake under 500 and upper 3500 kcal [17] and those who did not report lunch or dinner.

Food and beverage items were categorized into groups based on the NOVA food classification system, considering the nature, extent, and purpose of industrial processing [18,19]. This classification system offers a comprehensive framework by classifying food items into four main groups according to the level and intention of their industrial processing rather than their nutritional value. (1) Unprocessed or minimally processed foods: this group includes natural foods that have undergone minimal processing such as cleaning, chilling, and pasteurization (e.g., fresh fruits, vegetables, nuts, and meats); (2) Processed culinary ingredients: these are derived from unprocessed or minimally processed foods through processes like pressing or refining and are used for food preparation (e.g., oils, fats, sugar, and salt); (3) Processed foods: this category encompasses foods that have been modified through methods such as adding salt, sugar, or oil to enhance their shelf life or taste while retaining the basic identity and nutritional properties of the original food (e.g., canned vegetables, fruits in syrup, cheeses, and freshly made bread); (4) Ultra-processed foods (UPFs): UPFs result from extensive industrial processes, often containing additives and preservatives and little to no whole foods (e.g., soft drinks, packaged snacks, reconstituted meat products, and pre-prepared frozen meals).

According to the NOVA classification system, we have identified 498 foods, 149 in NOVA 1, 14 in NOVA 2, 90 in NOVA 3, and 245 in NOVA 4. To calculate the percentage of energy from each NOVA group, we divided the energy content of each group by the total energy intake.

A sweet snacks group was created with all identified foods that contained: added sugar; honey, molasses and syrup; fruit jams, jams and marmalade; candies and gummies; chocolates and chocolate snacks; ice creams; sweet desserts; cakes (cakes, pies, croissants, etc.); and commercial biscuits and biscuits. Additionally, three variables were created to describe the consumption of sweet snacks across different meals: breakfast; morning and afternoon snacks; and dinner and evening snacks.

### 2.3. Sleep Quality Assessment

To assess the sleep habits of the children participating in our study, we utilized the Portuguese Children’s Sleep Habits Questionnaire (CSHQ-PT). The CSHQ-PT is a validated parent-reported instrument specifically adapted for Portuguese-speaking populations. It is designed to evaluate various dimensions of sleep habits in children aged 4 to 10 years, providing a comprehensive overview of sleep-related issues. The score of the questionnaire (sleep habits score) was calculated by summing the 33 items. Higher scores indicate a higher frequency of sleep problems [20]. The cutoff point of 48 or higher was defined to identify problem sleepers and non-problem sleepers (<48) [21].

### 2.4. Anthropometric Measurements

At their schools, participants’ height and weight were measured by trained researchers using standardized methods. The measurements were taken with the participants barefoot and dressed in light clothing. Weight was measured with a pediatric scale (SECA 799) and noted to the nearest 100 g. Body mass index was calculated and then converted into standard deviation (SD) scores, adjusted for age and sex, using the World Health Organization growth reference [22].

### 2.5. Sociodemographic Characteristics

Sociodemographic data were collected, including the child’s sex and birth date. The parent’s highest level of education was self-reported and categorized into three groups: elementary (≤9 years), high school (10–12 years), and higher education (>12 years). Additionally, household income was evaluated and divided into four categories: less than 1000, 1001–1500, 1501–2000, and over 2001 euros per month.

### 2.6. Statistical Analysis

The participants’ characteristics are presented for the whole sample and by sex as absolute and relative frequencies for categorical variables and as mean ± standard deviation for normal-distributed continuous variables. Skewness and kurtosis coefficients were used to check the normality of continuous variables.

Differences between groups were assessed using the student’s *t*-test for independent and continuous normally distributed variables and the Chi-squared test for categorical variables.

Generalized linear models were performed to investigate the association between the proportion of total energy intake, according to food processing group (unprocessed or minimally processed; culinary ingredients; processed foods; ultra-processed foods), sweet snack consumption (total and per meal), and sleep habits score.

Binary logistic regression models were employed to estimate the associations between sleep habits (outcome), the total energy intake of NOVA groups, and sweet snack consumption (predictors).

For all analyses, a crude univariate and multivariate model adjusted for age, sex, household income, and the highest parent’s education level was conducted.

The data are expressed as regression coefficients (B) in generalized linear model and odds ratio (OR) in binary logistic, with their respective 95% confidence intervals (CI 95%) and *p*-values. All analyses were performed using the Statistical Package for the Social Sciences (SPSS, Inc. Chicago, IL, USA, version 29.0), with a significance level of *p* < 0.05.

## 3. Results

The main characteristics of participants (*n* = 137) are presented in Table 1. The mean age of children was 7.9 ± 1.2 years, and 52.6% (*n* = 72) were girls. As can be seen, 40.2% of the children presented weight above the normal, 26.3% had overweight and 13.9% had obesity, with a higher prevalence in boys (18.5%).

The mean total energy intake (TEI) per day was 1967 ± 531 kcal, with higher values observed in boys (2050 ± 540 kcal) than in girls (1892 ± 515 kcal).

In addition, the mean intake from children’s diet for unprocessed or minimally processed foods (NOVA 1) was 49.6%, culinary ingredients (NOVA 2) was 2.2%, processed foods (NOVA 3) was 21.5%, and ultra-processed foods (NOVA 4) was 26.7%.

In terms of sweet snacks, children consumed around 76.3 ± 65.0 g per day, with higher consumption in the girl’s group (78.7 ± 70.7 g).

The mean sleep habits score was 45.6 ± 6.5 and 45.4 ± 7.2 for girls and boys, respectively, and 35.9% of children were classified as problem sleepers.

**Figure 1:**
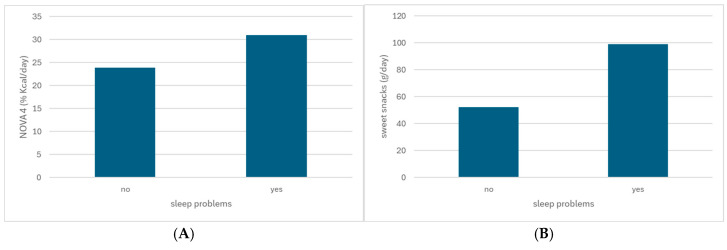
Consumption of NOVA 4 foods (**A**) and sweet snacks (**B**) among children with and without sleep problems.

Figure 1A,B illustrate, respectively, the consumption of NOVA 4 foods (31.0 vs. 23.9%) and sweet snacks (99.1 vs. 52.2 g) among children with and without sleep problems.

Additionally, parent’s education level and household income were also inquired, with most having completed high school (39.1%) or a higher degree (47.3%), and 35% having an income of 1001–1500 €.

Finally, no significant differences were observed between girls and boys across all analyzed variables.

Table 2 presents the association between energetic contribution to total energy intake of NOVA groups, sweet snack consumption, and sleep habits score.

For each increase of 1% of TEI in the consumption of foods from the NOVA 4 group, there was a significant increase of 0.115 (95% CI: 0.007; 0.224) in the sleep habits score, only in the non-adjusted model. Regarding sweet snack consumption and sleep habits score, the unadjusted and adjusted results, also in Table 2, revealed that for each increase of 1 g in the total daily consumption, the sleep habits score increased by 0.034 (95% CI: 0.010; 0.058). However, no significant associations were found between sweet snack consumption per meal and sleep habits score. Finally, when the model was adjusted, the association remained significant (B = 0.035; 95% CI: 0.011; 0.059).

The association between energetic contribution to the total energy intake of NOVA groups and sweet snack consumption and children’s sleep quality is presented in Table 3. Children with a higher energy contribution of ultra-processed foods (NOVA 4) had higher odds of achieving poor sleep quality (OR: 1.042; 95% CI: 1.004; 1.081). After adjustment for sex, age, parent’s education level, and household income, the same significant association was observed (OR: 1.043; 95% CI: 1.004; 1.084).

As shown in Table 3, an increased total daily consumption of sweet snacks (OR: 1.015; 95% CI: 1.005; 1.025), especially in the morning and afternoon snacks (OR: 1.017; 95% CI: 1.002; 1.031), is associated with a higher likelihood of having a lower sleep quality. These results remained significant after adjustment for all the confounders previously mentioned (OR: 1.016; 95% CI: 1.006; 1.026) and (OR: 1.018; 95% CI: 1.002; 1.033).

## 4. Discussion

The study identified a positive association between the sleep habits score and daily sweet snack consumption. Additionally, a positive link was observed between problem sleepers and the percentage of total energy intake from ultra-processed foods, as well as the total daily consumption of sweet snacks, particularly during morning and afternoon snack times. As far as we know, very few studies have reported whether these two dietary factors are associated with the sleep quality of children aged 5 to 11. In accordance with our findings, and despite the differences in population for outcome assessment, other studies have suggested the adverse impact of a diet high in ultra-processed and sugary foods on sleep quality [23,24].

A systematic review conducted to evaluate the association between dietary patterns and sleep among children and adolescents verified that long sleep duration and better sleep quality were consistently associated with healthy dietary patterns [25]. A previous study found that children who reported difficulty waking up in the morning tended to consume energy-rich foods more frequently and nutrient-dense foods less frequently [26]. This study also showed that children who consumed energy-rich foods were more likely to report feeling tired during the day. Chaput et al. [27] analyzed the association between sleep efficiency and dietary pattern score in children aged 9 to 11 and found that shorter sleep duration and poorer sleep efficiency were associated with unhealthy eating patterns. Moreira et al. [28] identified eight patterns from an 86-item FFQ in children aged 5–10 and established the link that sleep duration is negatively associated with fast food, sugar-sweetened beverages, and pastries.

Despite the fact that none of the studies mentioned specifically refer to “ultra-processed” or “NOVA 4” given that these food groups are products of extensive industrial processes and contain minimal to no whole foods, it is plausible to suggest that they could be classified as such [18].

Our analysis revealed that 26.7% of the children’s dietary energy came from NOVA 4, which is lower than in other populations. In a Brazilian sample, UPFs contribute approximately 48% of the total energy intake at age 8 [29], while in Belgian children aged 3 to 9, UPFs contribute around 33% [30]. A previous study, with subjects who were the same age or younger than ours, followed the same classification for food and used data from the Generation XXI cohort, found a high energy contribution from NOVA 1 + NOVA 2 (59.8%), followed by NOVA 4 (29.3%) and NOVA 3 (10.9%). In comparison, our results showed a similar energy contribution from NOVA 1 + NOVA 2 and UPF (51.8 and 26.7%, respectively) [4].

Due to advances in food production systems and changes in lifestyle trends, the consumption of ultra-processed foods, including sugary products, has been increasing in Portugal. This has led to a high prevalence of excessive sugar intake among Portuguese children [31]. About 72.4% consume sweet snacks such as candies, chocolates, chewing gums, chocolates, or lollipops up to three times a week [32]. In our research, the average daily intake of this food group was 76.3 g, identical to the average reported by the National Food, Nutrition, and Physical Activity Survey 2015–2016, conducted in Portugal, which also recorded 76.3 g and included sweets, candies, cakes, and breakfast cereals [33]. Although our sample may not fully represent the broader population, these findings indicate a consistent trend in sugar consumption within this age group.

When analyzing the sleep habits of the evaluated children, we found that 35.9% had poor sleep quality, with a mean score of 46.0. This finding is similar to Silva et al., whose mean score was 45.1 [20].

In our study, those with a higher energy contribution from NOVA 4 and higher consumption of sweet snacks had poor sleep quality, these findings are aligned with global research on this topic [1]. Similar patterns were noted for the sleep habits score, where an increased daily intake of sugary products, particularly during morning and afternoon snacks, was linked to a higher likelihood of a higher score, indicating poorer quality. According to this, other studies found that fast food, sugary drinks, and baked goods were negatively associated with sleep duration and sleep efficiency [27,28].

It is important to acknowledge that the literature presents mixed findings. Conversely, other research, conducted by Watson et al. [34], concluded that total dietary sugar consumption did not influence sleep in children.

The relationship between these dietary factors and the quality of children’s sleep may be explained by several mechanisms. For instance, foods containing high amounts of sugar might cause rapid fluctuations in blood sugar levels, which could potentially increase wakefulness and reduce sleep efficiency in children [14]. Moreover, having a diet high in UPFs can lead to nutritional deficiencies that may affect sleep. UPFs are generally low in essential nutrients such as magnesium, vitamin B6, and vitamin D, which have been associated with sleep issues, and a diet lacking these nutrients can disrupt the body’s production of melatonin, a hormone that regulates sleep [15]. Also, increased consumption of UPFs might be associated with heightened inflammatory responses due to chemical additives and the nutritional composition of these foods. Elevated levels of inflammatory markers such as C-reactive Protein (CRP) and interleukin-6 (IL-6) have been hypothesized to contribute to sleep disturbances [35].

While these mechanisms are not directly measured in our study, they are significant because sleep deprivation has been related to higher rates of overweight and obesity, increased body fat, and elevated hunger and appetite levels [36].

To our knowledge, this is the first report addressing the associations between processed food consumption and sleep quality in vulnerable contexts. Nevertheless, it is important to recognize the study’s limitations. This is a cross-sectional study, which does not allow for establishing causal relationships. The findings, supported by biological plausibility, generate hypotheses but cannot rule out reverse causality between sleep and diet. Some children provided only one 24 h recall, yet the average total energy intake did not significantly differ from those who provided two recalls. Additionally, there were missing responses to the sleep habits questionnaire, as well as information on parent’s education and household income, resulting in some missing data in our study. Despite controlling for sex, age, parent’s education level, and household income in our analyses, the relatively small sample size restricts our ability to include a wider range of socioeconomic factors and other variables. Although the sample size of our study limits the generalizability of the results, it represents an important effort within these age groups and specific contexts. Additional tests were conducted to confirm the strength and statistical significance of our results (see Supplementary Materials). Children with sleep problems have a higher consumption of sweet snacks (r = 0.383; *p* < 0.001) and a larger contribution from NOVA 4 to their daily energy intake (r = 0.253; *p* = 0.025). The Mann–Whitney test results support these findings, showing a significant difference for sweet snacks (U = 354, *p* < 0.001) and NOVA 4 contribution (U = 480, *p* = 0.022).

While the study primarily focused on this particular context, we recognize that the findings may vary in more affluent or geographically diverse populations. Future research should consider incorporating such diverse settings to examine the influence of socioeconomic factors and enhance the generalizability of the outcomes.

A significant strength of our research is the application of the Children’s Sleep Habits Questionnaire, which has been validated for Portugal and possesses suitable psychometric characteristics for screening sleep problems in children from 2 to 10 years old [20].

The NOVA classification system, though sometimes subject to debate, was employed in our study because it enables the identification of dietary patterns according to both the types of food and their degree of processing [37]. Finally, we assessed not only the overall daily intake of sweet snacks but also their consumption at individual meals, which led us to find a positive association with poor sleep quality. Such an approach can contribute to the development of other studies that assess causality between food consumption according to the degree of processing and sleep quality.

In light of our findings, we propose several recommendations to address these issues at multiple levels. Encouraging parents to reduce their children’s consumption of ultra-processed foods and to focus on providing balanced, nutritious meals can help improve sleep quality. Schools can play a key role by implementing nutrition education programs focused on the impact of dietary habits on sleep and by ensuring access to healthier food options, such as fruits, vegetables, and whole grains. Policymakers should consider supporting regulations to limit the availability of ultra-processed snacks in schools and developing guidelines that promote healthier dietary patterns. These interventions, alongside food literacy programs and initiatives to incentivize the purchase of fresh produce, could help reduce the consumption of ultra-processed foods and mitigate their negative effects on children’s sleep and overall health.

## 5. Conclusions

The results indicate that a higher intake of ultra-processed foods and sugary products, particularly during morning and afternoon snacks, is associated with poorer sleep quality. To improve children’s sleep health and overall well-being, it seems important to address their dietary habits. This study reinforces the importance of adopting healthy snacks, with the family and school playing a key role. Additionally, it raises awareness about the importance of limiting the consumption of ultra-processed foods in the school context, which will consequently contribute to healthy development during childhood.

## Figures and Tables

**Table 1:** Summary of participants’ characteristics by sex.

Variables	Total	Girls	Boys	*p*-Value
Age, mean ± SD	7.9 ± 1.2	7.9 ± 1.2	7.8 ± 1.1	0.726 ^1^
Weight status, *n* (%)				
Underweight	1 (0.7)	1 (1.4)	0 (0)	
Healthy weight	81 (59.1)	40 (55.6)	41 (63.1)	
Overweight	36 (26.3)	24 (33.3)	12 (18.5)	0.112 ^2^
Obesity	19 (13.9)	7 (9.7)	12 (18.5)	
TEI (kcal/day), mean ± SD	1967 ± 531	1892 ± 515	2050 ± 540	0.082 ^1^
Sweet snacks (grams), mean ± SD	76.3 ± 65.0	78.7 ± 70.7	73.7 ± 58.5	0.651 ^1^
NOVA Groups (% TEI), mean ± SD				
NOVA 1	49.6 ± 14.7	49.3 ± 15.2	49.9 ± 14.2	0.817 ^1^
NOVA 2	2.2 ± 2.6	2.2 ± 2.6	2.1 ± 2.5	0.926 ^1^
NOVA 3	21.5 ± 11.6	20.7 ± 11.0	22.4 ± 12.3	0.403 ^1^
NOVA 4	26.7 ± 14.4	27.8 ± 15.4	25.6 ± 12.4	0.363 ^1^
Sleep habits score, mean ± SD	46.0 ± 6.8	45.6 ± 6.5	45.4 ± 7.2	0.458 ^1^
Missings	59	30	29	
Sleep habits, *n* (%)				
Non-problem sleepers	50 (64.1)	25 (59.5)	25 (69.4)	
Problem sleepers	28 (35.9)	17 (40.5)	11 (30.6)	0.363 ^2^
Missings	59	30	29	
Parent’s education level, *n* (%)				
Elementary	15 (13.6)	8 (13.8)	7 (13.5)	
High School	43 (39.1)	22 (37.9)	21 (40.4)	0.965 ^2^
Bachelor’s degree or higher	52 (47.3)	28 (48.3)	24 (46.2)	
Missings	27	14	13	
Household income, *n* (%)				
<1000	22 (22.0)	12 (54.5)	10 (45.5)	
1001–1500	35 (35.0)	17 (48.6)	18 (51.4)	0.408 ^2^
1501–2000	26 (26.0)	12 (46.2)	14 (53.8)	
>2001	17 (17.0)	12 (70.6)	5 (29.4)	
Missings	37	19	18	

^1^ Independent sample *t*-test was used for continuous normally distributed variables; ^2^ X^2^-square test was used for categorical variables; Notes: % percentage.

**Table 2:** Association between energetic contribution to total energy intake of NOVA groups, sweet snack consumption, and sleep habits score.

Variables	Unadjusted Analysis		Adjusted Analysis *	
	B (95% CI)	*p*-Value	B (95% CI)	*p*-Value
NOVA Groups (% TEI)				
NOVA 1	−0.082 (−0.190; 0.025)	0.132	−0.094 (−0.198; 0.011)	0.080
NOVA 2	−0.266 (−0.817; 0.286)	0.345	−0.157 (−0.711; 0.396)	0.577
NOVA 3	−0.030 (−0.170; 0.109)	0.670	0.002 (0.136; 0.140)	0.979
NOVA 4	**0.115 (0.007; 0.224)**	**0.036**	0.106 (−0.002; 0.140)	0.055
Sweet snacks (g)				
Total	**0.034 (0.010; 0.058)**	**0.006**	**0.035 (0.011; 0.059)**	**0.005**
Breakfast	0.033 (−0.302; 0.098)	0.322	0.051 (−0.015; 0.117)	0.127
Morning and Afternoon snack	0.033 (−0.008; 0.740)	0.114	0.035 (−0.007; 0.076)	0.100
Dinner and Evening snack	0.023 (−0.025; 0.700)	0.347	0.025 (−0.023; 0.073)	0.306

Generalized linear models were employed to estimate the associations between sleep habits score, the total energy intake of NOVA groups, and sweet snack consumption. Notes: %—percentage; B—coefficient. * Adjusted for sex, age, highest parent’s education level, and household income.

**Table 3:** Association between energetic contribution to total energy intake of NOVA groups, sweet snack consumption, and sleep habits.

Variables	Unadjusted Analysis		Adjusted Analysis *	
	Problem Sleepers OR (95% CI)	*p*-Value	Problem Sleepers OR (95% CI)	*p*-Value
NOVA Groups (%TEI)				
NOVA 1	0.970 (0.937; 1.005)	0.096	0.968 (0.933; 1.004)	0.078
NOVA 2	0.893 (0.735; 1.085)	0.254	0.882 (0.720; 1.082)	0.229
NOVA 3	0.992 (0.950; 1.036)	0.728	0.996 (0.953; 1.042)	0.871
NOVA 4	**1.042 (1.004; 1.081)**	**0.031**	**1.043 (1.004; 1.084)**	**0.032**
Sweet snacks (g)				
Total	**1.015 (1.005; 1.025)**	**0.002**	**1.016 (1.006; 1.026)**	**0.003**
Breakfast	1.011 (0.992; 1.032)	0.262	1.013 (0.992; 1.034)	0.224
Morning and Afternoon snack	**1.017 (1.002; 1.031)**	**0.023**	**1.018 (1.002; 1.033)**	**0.022**
Dinner and Evening snack	1.013 (0.997; 1.029)	0.102	1.012 (0.997; 1.028)	0.118

Binary logistic regression models were employed to estimate the associations between sleep habits, the total energy intake of NOVA groups, and sweet snack consumption. Notes: %—percentage. * Adjusted for sex, age, parent’s education level, and household income.

## Data Availability

All data generated or analyzed during this study are available from the corresponding author on reasonable request. The data are not publicly available due to ethical reasons.

## Supplementary Materials

The following supporting information can be downloaded at: https://www.mdpi.com/article/10.3390/nu17020233/s1, Table S1: Pearson Correlations; Table S2: Mann-Whitney Test.
