# Supplementary material for: Processed Food Consumption and Sleep Quality in School-Aged Children: Insights from a Cross-Sectional Study
Source: Nutrients. 2025 Jan 10;17(2):233. doi: 10.3390/nu17020233 (PMC11767385; doi:10.3390/nu17020233)
Supplement: Supplementary file 1 [file nutrients-17-00233-s001.zip › nutrients-3396518-supplementary.pdf]

Supplementary Material

Table S1. Pearson Correlations

Variables Analyzed	Correlation Coefficient (r)	p-value	n
Sleep problems and sweet snacks	0.383	<0.001	78
Sleep problems and NOVA 4 contribution	0.253	0.025	78

Table S2. Mann-Whitney Test

Compared Group<br>(1 - non-sleep problems vs 2 - sleep problems)	U Statistic	p-value	Sample Size
Sweet snacks	354	<0.001	1 (50); 2 (28)
NOVA 4 contribution	480	0.022	1 (50); 2 (28)
